# What are the demands of telegeriatrics medical services for elderly patients during the COVID-19 pandemic

**DOI:** 10.3389/fpubh.2022.935684

**Published:** 2022-08-08

**Authors:** Yu Gong, Jianyuan Zhou

**Affiliations:** ^1^Department of Internal Medicine, Telemedicine Center, Shanghai Municipal Eighth People's Hospital, Shanghai, China; ^2^Department of Internal Medicine, Division of Nephrology, Shanghai Municipal Eighth People's Hospital, Shanghai, China

**Keywords:** telegeriatrics, medical services, family physicians, elderly patients, COVID-19

## Abstract

**Purpose:**

Elderly patients are associated with a higher risk of nosocomial cross infection during the COVID-19 pandemic. Providing medical services and primary care for elderly patients is a worldwide challenge. A new telegeriatrics system was established to provide medical services and primary care for elderly patients treated by family physicians. This study aimed to describe the operation mechanism of the new system and investigate the demands of telegeriatrics medical services for elderly patients treated by family physicians during the COVID-19 pandemic.

**Methods:**

A total of 1,353 elderly patients (aged≥60) treated by family physicians were enrolled. The proportion of the top 10 diseases of elderly patients applying the new system was analyzed. Differences in main diseases between elderly patients applying telegeriatrics medical services and outpatients in hospitals were compared. Differences between the new telegeriatrics system in our study and telemedicine systems of other studies in other countries were analyzed.

**Results:**

Constituent ratios of chronic kidney disease, type 2 diabetes mellitus, and coronary heart disease have the highest rate in elderly patients applying the new telegeriatrics system. Digestive diseases, cardiovascular diseases, and neurology diseases were the top three diseases of elderly outpatients.

**Conclusion:**

This is the first time that a new telegeriatrics system has been applied to provide medical services for elderly patients treated by family physicians during the COVID-19 pandemic. Chronic kidney disease, Type 2 diabetes mellitus, and coronary heart disease were found to be the top three diseases of elderly patients applying telegeriatrics medical services during the COVID-19 pandemic, which were different from the outpatients in general hospitals. The new telegeriatrics system guarantees elderly patients get equal rights to medical services. Results will provide a basis for the government health administrative department to formulate new telegeriatrics policies for elderly patients.

## Introduction

Elderly patients are associated with a higher risk of nosocomial cross infection during the COVID-19 pandemic. Providing medical services and primary care for elderly patients is a worldwide challenge. The great value of telegeriatrics medical services in the treatment of ailments in elderly patients treated by family physicians has attracted increasing attention during the COVID-19 pandemic. More importantly, inequities of the elderly in accessing telemedicine care have become the most urgent problem that seriously affects the health status and quality of life of elderly patients during the COVID-19 pandemic (1).

Telegeriatrics is the use of telemedicine to give elderly adults access to a geriatric specialist, which can provide medical services and primary care for elderly patients. Research on patient characteristics associated with telemedicine access for primary and specialty ambulatory care during the COVID-19 pandemic has been carried out (1). Evaluation of the efficiency, cost, and patients' and caregivers' acceptance of the telegeriatrics system in the older population had been conducted (2, 3). Research on the feasibility, acceptability, and cost-effectiveness in providing telemedicine to elderly patients have been carried out (4–6). The importance of the telegeriatrics system in the diagnosis and treatment of ailments in elderly patients has attracted ever-increasing attention.

As a megacity with more than 5.33 million elderly populations, it is of great significance to carry out research on telegeriatrics for the elderly during the COVID-19 pandemic in Shanghai. In a telemedicine center of a university-affiliated hospital, a new telegeriatrics system that can provide telegeriatrics medical services for elderly patients has been established. The key feature of the new system is that it is jointly conducted practice by family physicians in the Community Health Service Center and specialists in the university teaching hospital. It can provide the telegeriatrics medical services for elderly patients in the communities where they live. Unlike the typical telemedicine that has been practiced in other countries, the new system can provide a solution for the major issues in telemedicine where a doctor is unable to conduct a direct physical examination and the associated potential diagnostic error. The second is the new system is based on the multiple clinical disciplines in the large-scale public general hospital that can provide tele-diagnosis and tele-treatment of multiple clinical medical disciplines, including providing multiple clinical tele-diagnosis and tele-treatment services for elderly patients suffering from several diseases. It can provide medical services for several elderly patients at the same time. The third is that it is supervised by the government health administrative department that ensures the quality of medical services. The operation and maintenance of the new telegeriatrics system are funded by the government health administrative department, which can provide free medical services for elderly patients. The new telegeriatrics system provided convenient, efficient, and free medical services for the elderly. However, the demands for telegeriatrics medical services for elderly patients treated by family physicians are not well known. This study is to describe the operation mechanism of the new telegeriatrics system and investigate demands of telegeriatrics medical services and primary care for elderly patients treated by family physicians during the COVID-19 pandemic.

## Methods

In a telemedicine center of a university-affiliated hospital, an observational study was conducted. A consecutive sample of 1,353 elderly patients (aged≥60) applying for medical services *via* the new telegeriatrics system was investigated. Of which, 589 were male and 764 were female. As a control, 304,674 elderly outpatients in the university affiliated hospital were also investigated. All diseases of patients were classified according to the 10^th^ edition of the International Classification of Diseases. Each patient was classified according to the first diagnosis of the elderly patient applying medical services *via* the new telegeriatrics system. The proportion of the top 10 diseases of the elderly patients applying telegeriatrics medical services for the first time was analyzed. Gender, disease diagnosis, and disease ranking of all patients were recorded and analyzed. Differences of the main diseases between elderly patients applying the telegeriatrics medical services and that in the outpatient department of the general hospital were compared. Differences between the new telegeriatrics system in our study and telemedicine systems of other studies in other countries were analyzed.

The new telegeriatrics system is a medical website. This system is based on broadband communication and Visionvera Real Time HD video exchange technology. It is managed and supervised by the health administrative department to ensure the quality of medical services. To protect the patient's personal information and privacy, a dedicated optical broadband was used as the connection channel between the telemedicine center of the university teaching hospital and 29 community health stations in five community health centers. In the process of tele-diagnosis and tele-treatment, specialists in the large general hospital and family physicians (general practitioners) in community health centers use computers or smartphones and tablets as teleconsultation tools.

**Figure 1:**
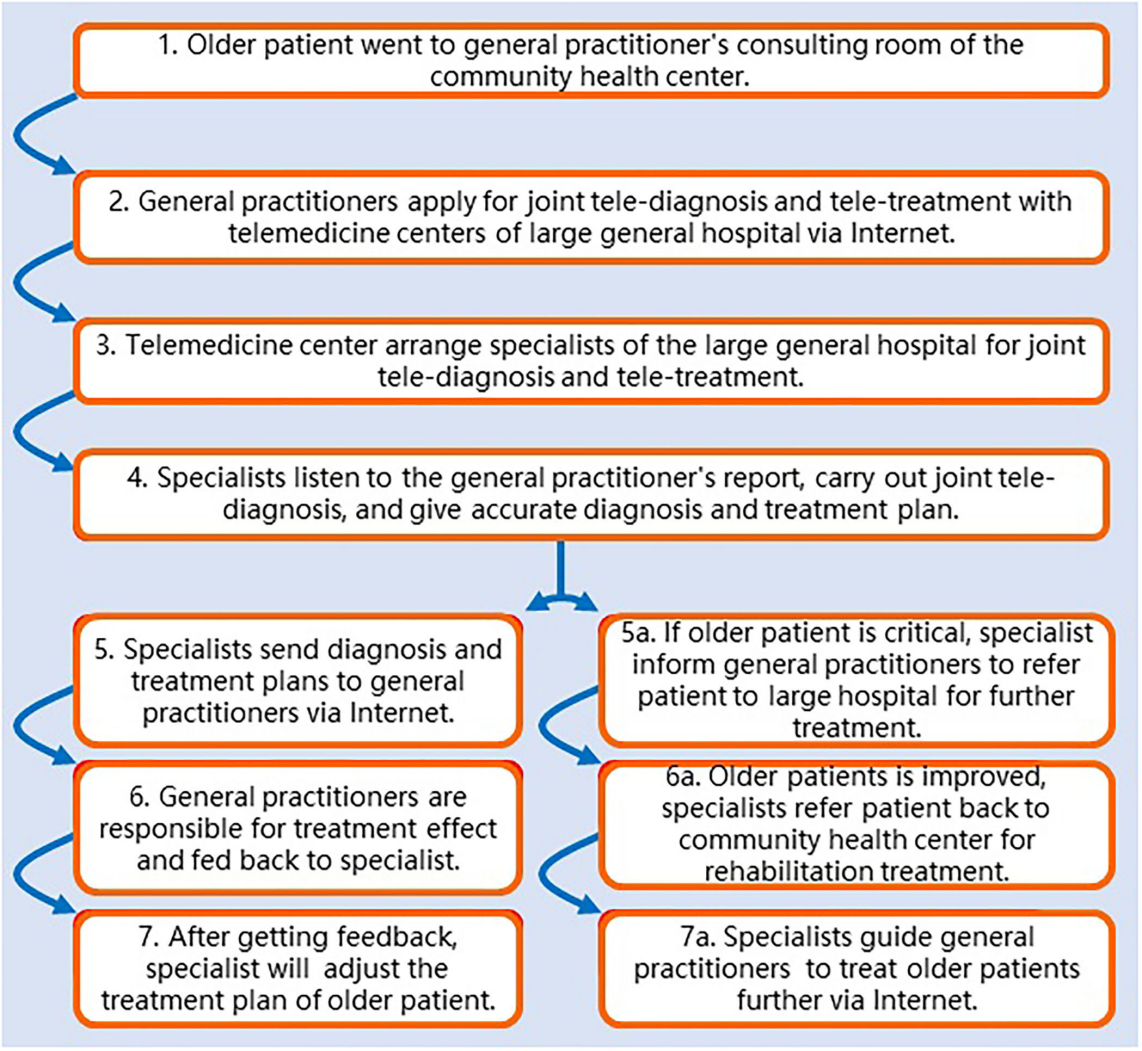
Flowchart of a new telegeriatrics system jointly conducted by family physicians in the Community Health Service Center and specialists in a university-affiliated hospital.

Geriatric specialists, cardiologists, nephrologists, endocrinologists, and gastro- enterologists of the university teaching hospital participated in the tele-diagnosis and tele-treatment to provide telegeriatrics medical services for elderly patients treated by family physicians. Elderly patients with heart disease, diabetes, kidney disease, nervous disease, digestive disease, etc. can receive tele-diagnosis and tele-treatment *via* the new telegeriatrics system. The process of the new telegeriatrics system is shown in chronological order in Figure 1. The process of the new telegeriatrics system is described in detail below:

1. Elderly Patients Go to the General Practitioner's Consulting Room of the Community Health Center Where They Live.

2. General Practitioners Apply for Tele-Diagnosis and Tele-Treatment From Experts of the Large General Hospitals Through the Internet.

3. Telemedicine Center Arranges Experts From Large General Hospitals to Carry out Joint Tele-Diagnosis and Tele-Treatment With General Practitioners.

4. Experts in Large General Hospitals Listen to the History Report of Elderly Patients From General Practitioners and Carry out Joint Tele-Diagnosis and Give Accurate Diagnosis and Treatment Plans to General Practitioners.

5. General Practitioners Carry out Treatment for Elderly Patients and Are Responsible for Evaluating the Treatment Effect and Feeding Back the Treatment Effect to Experts.

6. Experts From Large General Hospitals Will Adjust the Treatment Plan for Elderly Patients After Receiving Feedback on Treatment Effect.

7. General Practitioners Will be Informed of the new Treatment Plan Through the Internet and Apply the Modified Treatment Plan for Further Treatment.

If the condition of elderly patients is critical, experts from the large general hospitals will transfer the patients to a large general hospital for further treatment. After the elderly patients' condition was relieved. Experts from the large general hospitals will transfer the elderly patients back to the community health center for further rehabilitation treatment. In the follow-up treatment of elderly patients, experts from large general hospitals will guide general practitioners to do further treatment through the Internet.

The frequencies between the first diagnosis and follow-up visit:

After the first tele-diagnosis of elderly patients, family physicians (general practitioners) of the community health centers will bring elderly patients with chronic diseases into the “Family Sickbed Management System for Elderly Patients” for systematic chronic disease management. Then, family physicians will go to elderly patients' homes for home ward rounds twice a week. If the patient's condition deteriorated, the family physician will timely carry out joint tele-diagnosis and tele-treatment with experts in large general hospitals by using the portable iPad with wireless Internet in front of the bed of the elderly patient, so as to provide timely and accurate medical services for the elderly patient. This is called “mobile Internet-based bedside timely telegeriatrics for elderly patients.” The criteria for determining the eligibility for telegeriatrics medical service are:

1. The Condition of the Elderly Patient Was Significantly Aggravated in a Short Time.

2. The Condition of the Elderly Patients Changed, Requiring Computed Tomography (CT) or Magnetic Resonance Examination (MRI).

3. The Condition of the Elderly Patient Is Critically ill and Cannot be Solved by the Family Physician in the Community Health Center.

4. Family Physicians Encountered Severe Elderly Patients, Such as Elderly Patients With Acute Myocardial Infarction, Acute Cerebral Hemorrhage, Acute Renal Failure, Metabolic Acidosis, Hyperkalemia, Ketoacidosis, Acute Cerebral Infarction, Upper Gastrointestinal Hemorrhage, etc.

5. If the Patient's Condition Is Critical and Needs Urgent Care, We Will Immediately Send Experts of Corresponding Disciplines From Large General Hospitals to the Scene for Emergency Treatment.

By using the IBM SPSS Statistics Version 20.0 (IBM Inc., Chicago, USA), a statistical analysis of the study data was performed. *p*-value <0.05 was considered statistically significant.

The protocol for this research project has been approved by the Ethics Committee and the approval number is 2019-006. It conforms to the provisions of the Declaration of Helsinki in 1975 (as revised in Edinburgh 1983). Informed, written consent was obtained from every patient.

## Results

**Figure 2:**
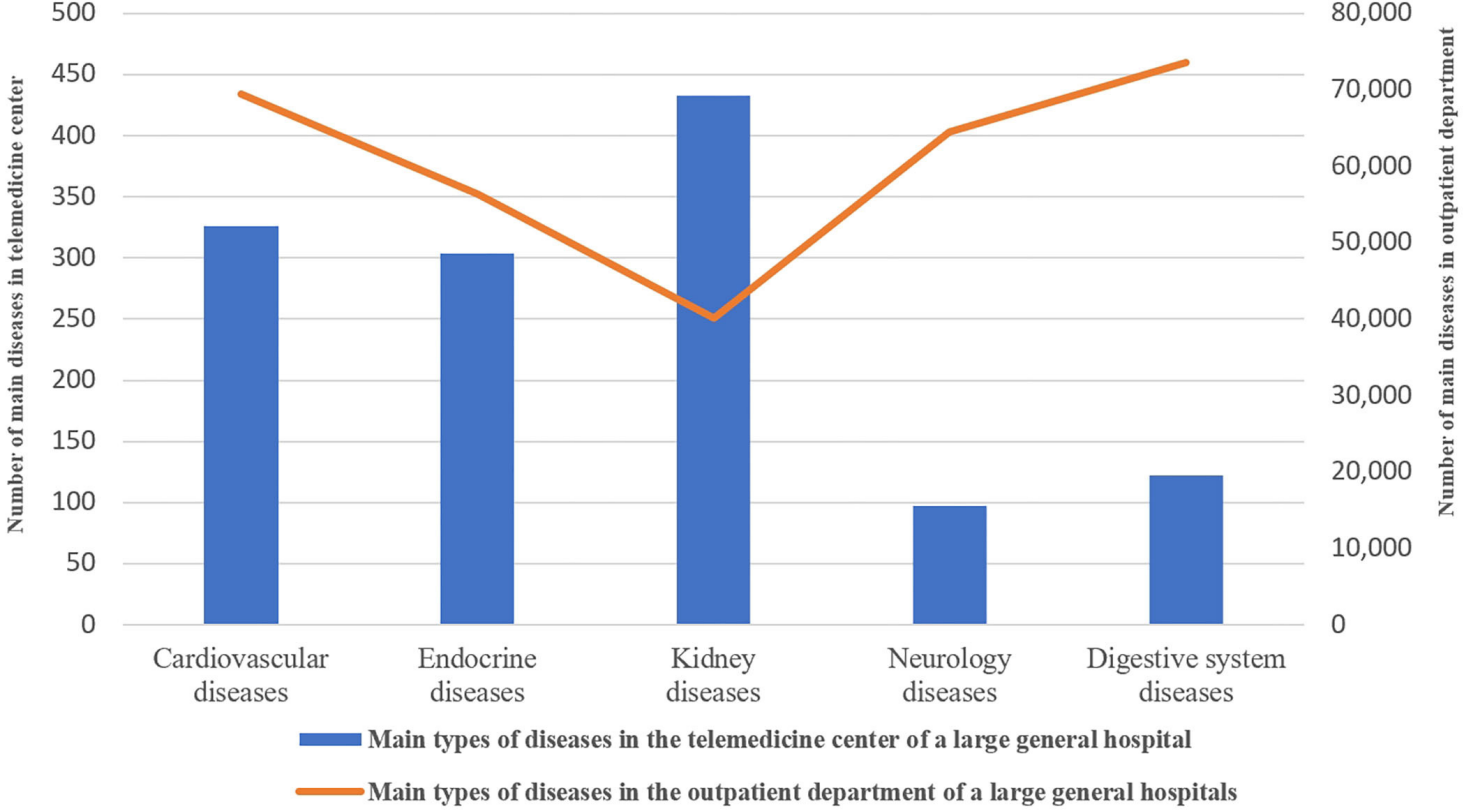
Differences between the main diseases of the elderly applying medical services *via* the new telegeriatrics system and the elderly outpatients in a university-affiliated hospital.

A total of 1,353 elderly patients applying medical services *via* the new telegeriatrics system were investigated. Among them, 589 were male elderly patients and 764 were female. The number of female elderly patients is higher than that of male elderly patients. In this study, 254 elderly patients with chronic kidney disease (CKD) out of 1,353 participants (18.77%), 184 elderly patients with Type 2 diabetes mellitus (13.60%), and coronary heart disease (11.90%) were found to have the highest rate in elderly patients applying for the medical services in the new telegeriatrics system during the COVID-19 pandemic. The constituent ratio of these three diseases was the highest, followed by hypertension, urinary tract infection, hyperuricemia, chronic gastritis, hyperlipidemia, hypothyroidism, and thyroid nodule. The constituent ratio of these seven diseases was 9.68, 5.24, 5.10, 4.51, 3.03, 2.29, and 2.14%, respectively (see Table 1). In this study, CKD, Type 2 diabetes mellitus, and coronary heart disease were found to be the top three diseases in elderly patients applying for medical services *via* the new telegeriatrics system during the COVID-19 pandemic. The main diseases of the elderly outpatients in the outpatient department of the general hospital were investigated. Digestive system diseases, cardiovascular diseases, and neurology diseases were found to be the top three diseases of elderly outpatients in the general hospital (see Figure 2).

**Table 1:** Constituent ratio of major diseases of elderly patients (≥60 years old) applying telegeriatrics services during the COVID-19 pandemic.

**Sort**	**Classification of diseases**	**ICD-10**	**Number of cases**	**Constituent ratio (%)**
1	Chronic kidney disease	N18.900	254	18.77
2	Type 2 diabetes mellitus	N18.900x005	184	13.60
3	Coronary heart disease	E11.900	161	11.90
4	Hypertension	I25.103	131	9.68
5	Urinary tract infection	I10.X00	71	5.24
6	Hyperuricemia	N39.000	69	5.10
7	Chronic gastritis	E70.001	61	4.51
8	Hyperlipidemia	K29.500	41	3.03
9	Hypothyroidism	E78.500	31	2.29
10	Thyroid nodule	E05.900	29	2.14

ICD-10, 10th edition of the International Classification of Diseases.

The differences between the new telegeriatrics system in our study and the telemedicine systems of other studies in other countries were analyzed. It was found that the telemedicine mode in our study is a joint telemedicine mode, which was jointly conducted by family physicians in the community health service center and specialists in large general hospitals. The tele-diagnosis and tele-treatment were supported by clinical multidisciplinary and it can provide tele-diagnosis and tele-treatment for patients with multiple complications. A doctor can conduct a physical examination on the patient to avoid misdiagnosis. The new telegeriatrics system in our study has realized the information sharing of the image of CT and MRI by family physicians in the community health service center and specialists in large general hospitals. A comparison of the new telegeriatrics system in our study and telemedicine systems of other studies in other countries are shown in Table 2.

**Table 2:** Comparison of the differences between the new telegeriatrics system in our study and telemedicine systems of the main studies mentioned in the discussion.

**Study**	**In the study of Gong Y, et al. (In this study)**	**In the study of Batsis JA, et al**.	**In the study of Egede LE, et al**.	**In the study of Goldstein CM, et al**.	**In the study of Maresca G, et al**.	**In the study of Batsis JA, et al**.
Telemedicine mode applying in different countries	Shanghai, China	Lebanon, NH, USA.	Charleston, SC, USA	Akron, Ohio, USA	Italy	Rouen, France
Clinical medicine discipline scale	Clinical multidisciplinary joint consultation	Single clinical medical discipline	Single clinical medical discipline	Single clinical medical discipline	Single clinical medical discipline	Single clinical medical discipline
Diseases of patients applying for telemedicine services	Chronic diseases of elderly patients	obesity manage- ment	non-inferiority of behavioral activation therapy for major depression	older adults with heart failure	elderly people with depressive symptoms	elderly patients with heart failure and dermat- ology
Patient's age	Elderly Patients (≥60 years old)	Elderly and non-elderly people	Veterans (aged ≥58 years) meeting DSM-IV criteria for major depressive disorder	Older adults average age was 69 years	Elderly people	Elderly patients
Telemedicine mode	Jointly conducted by family physicians in the community health service center and specialists in large general hospitals	A doctor and a patient use the internet alone for consultation	A doctor and a patient use the Internet alone for consultation	A doctor and a patient use the internet alone for consultation	A doctor and a patient use the internet alone for consultation	A doctor and a patient use the internet alone for consultation
Can consultation be conducted for patients with multiple complications?	Yes	No	No	No	No	No
Platform for telemedicine	Large general hospital with clinical multidisciplinary	A single clinical medicine discipline	A single clinical medicine discipline	A single clinical medicine discipline	A single clinical medicine discipline	A single clinical medicine discipline
Whether doctor can conduct physical examination on the patient to avoid misdiagnosis?	Yes	No	No	No	No	No
Information sharing of laboratory test results	Yes	No	No	No	No	No
Information sharing of imaging CT and MRI examination results	Yes	No	No	No	No	No
Whether mobile Internet telemedicine can be conducted?	Yes	No	No	No	No	No
Whether follow-up internet based home nursing services can be carried out?	Yes	No	No	No	No	No
Charge or not?	Free charge	Charge	Charge	Charge	Charge	Charge
Whether it is charitable?	Charitable	Non charitable	Non charitable	Non charitable	Non charitable	Non charitable

CT, Computed Tomography; MRI, Magnetic Resonance Imaging.

## Discussion

In this study, Chronic kidney disease, Type 2 diabetes mellitus, and coronary heart disease were found to be the top three diseases of elderly patients applying telegeriatrics medical services during the COVID-19 pandemic. The new telegeriatrics system in our study guarantees elderly patients get equal rights of medical services.

Equality of access to medical services for the elderly is very important. Due to the increase in age, it is difficult for the elderly to use the Internet and smart devices, which led to the inequality of elderly patients in accessing telemedical services and primary care. Research on the equality in accessing medical services and primary care from telemedicine has been carried out. In the United States, Eberly et al. found that elderly patients had lower rates of telemedicine use which indicates inequities in accessing telemedicine care for elderly patients, which warrants further attention (1). This may be due to the poor ability of elderly patients to use the Internet and smart devices. In our study, an elderly patient was initially examined and diagnosed by family physicians in the community health center. Family physicians will help elderly patients to contact specialists in large general hospitals *via* the Internet to carry out joint tele-diagnosis and tele-treatment with the specialist. This helps elderly patients overcome difficulties in using the Internet and smart devices. This is a useful attempt and innovation to solve the “digital gap of medical services” faced by the elderly. And the operation and maintenance of the new telegeriatrics system are funded by the government health administrative department, it can provide free medical services and primary care for elderly patients which overcomes the economic inequities in accessing telemedicine care.

There are some limitations of the study. This is a single-center study with a relatively small sample size of elderly patients and the observation time of elderly patients was relatively short. It is necessary to conduct a long-term observation study of multiple centers with larger samples in the future.

In clinical practice, the prevention of diseases in the elderly is more important than treatment. The new telegeriatrics system in our study provides medical services and primary care for elderly patients and overcome the disadvantage of currently used models of telegeriatrics. It is supervised by the government health administrative department to ensure medical services quality. The new telegeriatrics system is funded by the health administrative department so it can provide free medical services and primary care for elderly patients. Telegeriatrics may become the main way for elderly patients to seek medical services in the future. It is of great value to clarify medical service demands of telegeriatrics for elderly patients. An acquaintance of medical service demands of telegeriatrics for elderly patients may provide clinicians an opportunity to take timely measures to improve outcomes for elderly patients.

In this study, it was found the constituent ratio of CKD (formerly referred to as chronic renal insufficiency or chronic renal failure) was the top disease of elderly patients applying for telegeriatrics medical services. This result is obviously different from the main disease of elderly outpatients in general hospitals which were digestive system diseases and cardiovascular diseases. In the USA, it was found that the incidence rate of CKD increased dramatically in the elderly population from 1992 to 2005 (7). In our study, it was shown that the constituent ratio of CKD ranked the highest, accounting for 18.77% of elderly patients applying for medical services *via* the new telegeriatrics system. CKD refers to chronic renal structure and dysfunction caused by various reasons (the history of renal damage is more than 3 months), including normal and abnormal pathological damage of renal glomerular filtration rate (GFR), abnormal blood or urine composition, abnormal imaging examination, or unexplained decline of GFR (<60 ml/min·1.73 m^2^) for more than 3 months. An estimated 37 million adults in the United States may have CKD. The risk factors for CKD were old age, CKD family history, diabetes, hypertension, obesity-metabolic syndrome, high protein diet, hyperlipidemia, and hyperuricemia. It is very important to take the steps needed to protect kidney function when it is found early. Rapid improvement of living standards resulted in a significant increase in the morbidity of diabetes, hypertension, and hyperuricemia in elderly people. These in turn led to the corresponding increase in the morbidity of diabetic nephropathy, hypertensive nephropathy, and uric acid nephropathy. These diseases will eventually develop to the stage of chronic renal insufficiency which contributes to an increase in the incidence of CKD.

With the development of the aging of human society, the incidence of Type 2 diabetes mellitus and coronary heart disease gradually increased with the increase of the age of the elderly people. The dietary habits with higher content of sugar, fat, and cholesterol are major causes of coronary heart disease and diabetes mellitus. In recent years, the incidence of obesity in middle-aged people has increased significantly. As one of the main risk factors for Type 2 diabetes, obesity plays an important role in the increase in the incidence of Type 2 diabetes. In a national study with a nationally representative sample of 46,239 adults in China, it was found that the prevalence of diabetes of elderly adults (aged≥60) was 20.4% (8). These results indicate diabetes has become one of the major chronic diseases of the elderly in China. In the USA, cardiovascular disease, osteoporosis, and dementia are common chronic conditions at age 85 (9). This is the difference in major diseases of elderly adults between the United States and China.

In our study, it was found that digestive system diseases, cardiovascular diseases, and neurology diseases were the top three diseases of the elderly outpatients in the general hospital. This is mainly related to long-term unhealthy eating habits and the age growth of elderly people who often eat fried food and barbecue food. Fried food contains acrylamide and barbecue food contains nitrosamines. These harmful substances will lead to an increase in the incidence rate of digestive system diseases, which results in digestive system diseases being the main disease of elderly patients in the outpatient department.

It has been reported that telegeriatrics is feasible and acceptable in providing primary care and medical services to elderly patients, and clinicians should apply telegeriatrics in daily practice to overcome the barriers of distance and access to medical services (5). Telegeriatrics could close the gap for elderly patients who are unable to go to the hospital in time. Shah et al. found emergency care enhanced by telemedicine is an acceptable way to provide emergency care to the elderly in senior living communities (10). In our study, elderly patients accounted for the majority of applications of telegeriatrics medical services, which showed elderly patients have a great demand for telemedicine. The telegeriatrics system has been used to monitor elderly patients with high-risk chronic heart failure for an early evaluation of signs and symptoms of acute decompensation (3). The use of the telegeriatrics system as a telehealth intervention has been used to improve medication adherence in elderly patients with heart failure (11). Since the mode of telemedicine in other countries rely upon direct communication between a doctor and a patient *via* the Internet but the doctor would be unable to give the patient a direct physical examination, it may lead to diagnostic errors. In our study, the elderly patient was initially examined and diagnosed by family physicians in the community health center. If the patient's condition is complex, family physicians will contact a specialist in large general hospital *via* the Internet and carry out joint tele-diagnosis and tele-treatment with the specialist. Subsequently, the patient may be transferred to a large general hospital for further treatment. The new telegeriatrics system in our study provides a solution for the major issues in telemedicine where the consultation and diagnosis are conducted directly between one patient and one doctor alone through the Internet without being able to perform a physical examination of the patient, which may lead to difficulties in making a correct diagnosis, or even result in misdiagnosis.

The application of medical services *via* the telegeriatrics system in home health monitoring can help self-management of the elderly patients with chronic diseases. Recent studies have found that low adherence to recommendations is one of the causes of unsatisfactory results of chronic heart failure treatment in elderly patients (12). In our study, the medical orders for elderly patients were issued firstly by the specialists in the large general hospital and then supervised by family physicians. The Telegeriatrics system can greatly shorten the space distance that elderly patients need to cross and reduce the time it takes to visit a doctor. It was found that elderly patients are more satisfied with their medical services than younger patients and in the oldest cohorts, are at high risk for passive relationships and communication complications related to low literacy and poor health status (13). Family physicians should be familiar with the medical services demands of elderly patients.

Telemedicine has been successfully applied in some medical professions and can be used as a useful way to provide much-needed intensive behavioral therapy in remote and resource-poor environment (14). The way of applying medical services *via* the telegeriatrics system to serve patients' mental health can widen the channels of seeking medical treatment for elderly patients (15). In recent years, the incidence of various psychiatric illnesses has increased significantly in the elderly. The psychotherapy provided by the telegeriatrics system for senile depression patients was found to be superior to that provided in the physician's office and it can be used to overcome the nursing obstacles related to the distance of elderly patients (16). The use of the telegeriatrics system in monitoring and managing the mental health of elderly patients will be a new attempt in the application of telegeriatrics in the management of chronic diseases in elderly people.

Taddei et al. found hypertension was the most common reason for a medical appointment in elderly patients with cardiovascular disease (17). In our study, hypertension was found to be the fourth disease in elderly patients applying the new telegeriatrics medical services. Hypertension is one of the common chronic diseases in the elderly. With the increasing age of elderly patients, the incidence rate of hypertension also increased. Hypertension is an important risk factor for stroke and death in elderly patients. Therefore, family physicians should attach great importance to the treatment of hypertension in elderly patients. Strictly controlling the blood pressure of elderly patients can significantly reduce the stroke incidence rate and mortality of elderly patients with hypertension.

The telegeriatrics system has been widely used in the medical services of elderly patients in city communities and remote areas. Suburbs of American cities have difficulties to provide medical services to elderly adults and the use of the telegeriatrics system can help solve the problems of delivering healthcare and medical services to rural elderly patients (18). It has been reported that telegeriatrics can be an appropriate way to provide medical services to elderly patients more efficiently to prevent vascular diseases and other complications (19). Compared with medical services in urban areas, medical services in rural areas often faces problems, such as shortage of medical specialists, facilities with inferior equipment, and insufficient resources. In France, a telegeriatrics experiment was carried out to favor access to care for disabled elderly people (20). In our study, it was found that the new telegeriatrics system can provide convenient and efficient medical services for elderly patients, including the elderly living in remote suburban communities.

The telegeriatrics system can reduce medical expenditure and meet the growing demand for medical services for the elderly. In the new telegeriatrics system of our study, the cooperation of family physicians and specialists of a university teaching hospital made it possible that elderly patients can enjoy expert diagnosis and treatment in their families or in the communities where they live. After the joint tele-diagnosis and tele-treatment, elderly patients can refer to a large-scale general hospital for further treatment according to the recommendations of specialists. During the joint tele-diagnosis and tele-treatment, specialists and family physicians share information on patients' diagnoses and treatment through the Internet, including patients' medical history, health records, and laboratory test results. It can break the time and space constraints, and help realize the professional guidance and technical support to family physicians online. The practice of the new telegeriatrics system has proved it is an effective telegeriatrics mode to provide medical services for elderly patients.

Telegeriatrics has become a new way to improve the diagnosis and treatment level of family physicians in primary medical institutions and to meet the growing demand for medical services demands for elderly patients in the suburbs. The new system played an important role in the treatment of elderly patients with chronic kidney disease, type 2 diabetes mellitus, and coronary heart disease treated by family physicians and enabled elderly patients to receive timely treatment.

There are other benefits of elderly patients applying medical services *via* the telegeriatrics system. The potential contribution of applying medical services *via* the telegeriatrics system to the reduction of mortality or morbidity, and in reducing the occurrence of hospitalization is currently being evaluated and their impact on health economics is also being validated (21). In our study, due to the application of the new telegeriatrics system, timely diagnosis and treatment were achieved that may reduce the possibility of hospitalization of elderly patients.

In France, the economic and social benefits of telemedicine solutions had been studied and telemedicine projects concerning the elderly are highly valued (22). In our study, the new telegeriatrics system provides free medical services and primary care for elderly patients which overcomes the economic inequities in accessing telemedicine care. Therefore, the social benefits of the new telegeriatrics system far exceed economic benefits. We provide high-quality telegeriatrics medical services and primary care to elderly patients regardless of their gender, wealth, nationality, urban or suburban areas, and the severity of their condition. Providing telegeriatrics medical services and primary care to elderly patients is a charity.

We have done some research about experience globally and compared different cases according to various situations. We compared five different telemedicine systems in different countries including those in the United States, Italy, and France mentioned in the above discussion. The comparison of the new telegeriatrics system in our study and telemedicine systems of the main studies mentioned in the discussion can be seen in Table 2. It was found that the telemedicine system in our study was jointly conducted by family physicians in the community health service center and specialists in large general hospitals. The tele-diagnosis and tele-treatment were supported by clinical multidisciplinary and it can provide tele-diagnosis and tele-treatment for patients with multiple complications. A doctor can conduct physical examination on the patient to avoid misdiagnosis. The new telegeriatrics system has realized the information sharing of the image of CT and MRI examination results by family physicians in the community health service center and specialists in large general hospital. The telemedicine system in our study can provide follow-up Internet-based home nursing services and it does not charge patients any fees, which is charitable.

Mankind has entered an aging society and the Internet age. It is of great value to guarantee elderly patients get equal rights of medical services. Inequality of the elderly in telemedicine has become an important factor in the health and longevity in the elderly in an aging society. Timely solving this serious problem can greatly improve the quality of life and prolong the life of elderly patients.

The new telegeriatrics system in our study provides effective medical services and primary care for elderly patients. It is recommended to introduce and promote to other cities or regions to benefit elderly patients.

During the COVID-19 pandemic, it is of great significance to provide convenient and efficient telemedicine services to elderly patients. Elderly patients are susceptible to COVID-19 and the mortality of elderly patients with COVID-19 was significantly higher than that of patients of other ages. How to prevent elderly patients from being infected with COVID-19 is a very urgent and important problem. The new telegeriatrics system in our study provided convenient and efficient medical services to elderly patients to ensure that elderly patients not to go to crowded hospitals repeatedly. Elderly patients can receive tele-diagnosis and tele-treatment *via* the new telegeriatrics system to avoid the cross infection of COVID-19. Telegeriatrics may play an important role in the struggle to prevent elderly patients from being infected with COVID-19. It may become the main way for elderly patients to seek medical services in the future.

## Conclusion

This is the first time that a new telegeriatrics system has been applied to provide medical services for elderly patients treated by family physicians during the COVID-19 pandemic. Chronic kidney disease, Type 2 diabetes mellitus, and coronary heart disease were found to be the top three diseases of elderly patients applying telegeriatrics medical services during the COVID-19 pandemic that were different from the outpatients in a general hospital. The new telegeriatrics system in our study is different from the telemedicine systems in other countries. It guarantees elderly patients get equal rights of medical services. The results of this study will provide a basis for government health administrative department to formulate new telegeriatrics policies for elderly patients.

## Data availability statement

The raw data supporting the conclusions of this article will be made available by the authors, without undue reservation.

## Ethics statement

The studies involving human participants were reviewed and approved by Ethics Committee of Shanghai Municipal Eighth People's Hospital and the approval number is 2019-006. The patients/participants provided their written informed consent to participate in this study.

## Author contributions

YG and JZ helped to conceptualize the study and drafted the manuscript. YG played a key role in helping collect and analyze the data. All authors helped to revise the manuscript, contributed to the article, and approved the submitted version.

## Funding

This work was supported by the Shanghai Municipal Xuhui Medical Research Project Fund (grant number SHXH201957), which was sponsored by the Shanghai Municipal Xuhui Health Committee (Government Health Administrative Department).

## Conflict of interest

The authors declare that the research was conducted in the absence of any commercial or financial relationships that could be construed as a potential conflict of interest.

## Publisher's note

All claims expressed in this article are solely those of the authors and do not necessarily represent those of their affiliated organizations, or those of the publisher, the editors and the reviewers. Any product that may be evaluated in this article, or claim that may be made by its manufacturer, is not guaranteed or endorsed by the publisher.
